# Impact of Health Literacy on the Progression of Frailty after 4 Years among Community-Dwelling Older Adults

**DOI:** 10.3390/ijerph19010394

**Published:** 2021-12-30

**Authors:** Yasuyo Yoshizawa, Tomoki Tanaka, Kyo Takahashi, Mahiro Fujisaki-Sueda-Sakai, Bo-kyung Son, Katsuya Iijima

**Affiliations:** 1 Department of Healthy Life Expectancy, Graduate School of Medicine Juntendo University, Tokyo 113-0034, Japan; 2 School of Nursing, Tokyo Women’s Medical University, Tokyo 162-8666, Japan; 3 Institute of Gerontology, The University of Tokyo, Tokyo 113-8656, Japan; tmk-tanaka@iog.u-tokyo.ac.jp (T.T.); k.takahashi@iog.u-tokyo.ac.jp (K.T.); fujisam@iog.u-tokyo.ac.jp (M.F.-S.-S.); son@iog.u-tokyo.ac.jp (B.-k.S.); iijima@iog.u-tokyo.ac.jp (K.I.); 4 Department of Public Health, School of Medicine, Dokkyo Medical University, Mibu 321-0293, Japan; 5 Department of Public Health Nursing, School of Health Science, Tohoku University, Sendai 980-8575, Japan; 6 Institute for Future Initiatives, The University of Tokyo, Tokyo 113-8656, Japan

**Keywords:** community-dwelling older adults, frailty, health literacy, Kihon Checklist

## Abstract

Health literacy (HL) promotes healthy lifestyle behaviors among older adults, and its relationship with frailty remains unclear. This study examined whether HL is a predictor of frailty progression among community-dwelling older adults. Data from two surveys conducted in 2012 and 2016 involving older residents (mean age, 71.6 ± 4.6 years) of Kashiwa City, Chiba Prefecture, Japan were used. Only healthy individuals without frailty and cognitive impairments participated in the 2012 assessment, where the Kihon Checklist (KCL), HL, and other variables were assessed. Logistic and multiple logistic analyses were used to assess the effects of HL and other factors on frailty between the ‘high HL’ vs. ‘low HL’ groups in 2012 and between the ‘robust’ vs. ‘frailty-progressing’ groups in 2016. Of the 621 robust participants, 154 (25.4%) had progression of frailty in 2016, which was significantly associated with advanced age, higher KCL score, lower HL, poor mental health, and lack of social support. Furthermore, low HL was a predictor of frailty progression. Low HL may be associated with frailty progression. The obtained results suggest that increased health literacy should be effective in preventing frailty for community-dwelling older residents.

## 1. Introduction

In 2016, the reported average life expectancy in Japan was 80.9 years for men and 87.1 years for women [1]. In the same year, ‘healthy life expectancy’, which is defined as the maximum age people can maintain their active lifestyles without critical health issues and constant reliance on nursing care, was 72.1 and 74.7 years for men and women, respectively. Thus, there is a gap of 8.8 years for men and 12.3 years for women between actual life expectancy and healthy life expectancy; this implies that there are various restrictions in the activities of daily living of older adults [1]. Therefore, it is essential to prolong healthy life and effectively eliminate the gap between actual life and healthy life expectancy. Furthermore, as society is becoming an aging one, this is important not only for individuals’ quality of life and wellbeing but also for a sustainable social security system and economy. Thus, one of the goals of the Ministry of Health, Labour, and Welfare (MHLW) of Japan is to extend healthy life expectancy by at least three years by 2040 [2].

Frailty is a widely recognized concept that is generally applicable to the aged population with health issues; frailty can often cause vulnerability to external stresses in everyday lives, increase the need for constant nursing care, and as such, it is difficult to maintain independent and active lifestyles [3,4]. While the aging-related progression of frailty is a critical risk factor for healthy life expectancy, adequate intervention and support can maintain or improve the health functions necessary for active everyday living. A total of 11.5% of older adults aged 65 years and above are considered ‘frail’. Further, aging is a critical factor for impaired health, as reflected by the frailty rates of 5.6%, 7.2%, 16%, and 34.9% in older adults aged 65–69, 70–74, 75–79, and >80 years, respectively [5]. Therefore, it is necessary to identify factors for frailty progression and devise effective intervention strategies to prolong healthy life expectancy.

There is growing interest in research on health literacy (HL). HL is defined as the skill to collect, evaluate, and select health-related information from multiple sources [6]. As HL has been shown to drive positive behavioral changes, this finding is useful in instituting strategies to avoid negative everyday habits such as smoking and encourage healthy routines (e.g., exercise) [7]. In addition, the beneficial effects of HL have also been reported in patients with diabetes [8,9] and heart disease [10,11]. Greater HL indicates the higher knowledge about health and the ability to control medical expenses [12]. Similarly, the previous studies involving the older population have shown that higher HL is associated with optimum cognitive, physical, mental, and chronic health functions [13]. Several studies have reported that HL is a predictor of frailty progress, therefore leading to the prevention of frailty in older adults. However, most of those studies used an observational design [14,15], but longitudinal studies should also be required to further investigate causal relationships between HL and frailty.

Along with the widespread use of the Internet, health information has become more accessible; therefore, intervention strategies focusing on HL may be more efficient. According to the Ministry of Internal Affairs and Communications, approximately 90% of the entire population in Japan uses the Internet. Furthermore, the Internet usage rate is relatively high in the older population with 74.2% and 57.5% in the elderly in their 70s and 80s, respectively [16]. According to existing online information sources in this ‘information era’, HL may have a major impact on changing individual lifestyles to prolong healthy life expectancy.

The primary aim of this study was to investigate whether HL is a key determinant of frailty progression among community-dwelling older adults. To the best of our knowledge, this is the first study to employ a longitudinal design to monitor changes over four years among community-dwelling older adults. Findings from this study will provide implications for designing intervention strategies to improve HL for prolonged healthy life expectancy. 

## 2. Materials and Methods

### 2.1. Research Design and Participants 

This study assessed the factors for frailty progression by comparing the data obtained from surveys conducted in 2012 and 2016. The surveys were part of a prospective cohort study of community-dwelling older residents of Kashiwa City, Chiba Prefecture, Japan (the Kashiwa Study). Kashiwa City has a population of about 430,000 and is officially registered as a ‘core’ city. It is located in the northwestern part of Chiba Prefecture, which is adjacent to Tokyo and has functioned as the representative bed town for the Tokyo metropolitan area. The older population is increasing year by year, and the aging rate is currently 26.2% [17]. Of 80,000 older residents aged ≥65 years in Kashiwa City, invitations were mailed to randomly selected 12,000 participants of the Kashiwa Study. A total of 2044 older adults participated in the initial baseline survey conducted in 2012 in 12 local community centers and health institutions, with a male and female proportion of 56% and 44%, respectively. 

Anthropometric data (i.e., age, sex, height, weight, body mass index [BMI]), educational background, household patterns, and income status were obtained in 2012. Educational background was classified into three categories: (i) elementary and junior high school graduates, (ii) high school graduates, and (iii) university/junior college/vocational school graduates. Income status was classified into two categories: high and low, based on the Basic Resident Register.

**Figure 1:**
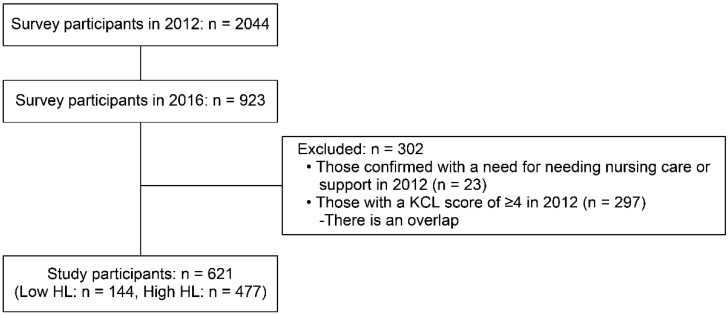
Data flow.

After 4 years, i.e., in 2016, from the initial survey, an invitation postcard was sent out to the participants, but due to factors such as moving, death, poor physical condition, hospitalization, and schedule mismatch, only 923 of them returned to undertake the second survey. However, 302 individuals were excluded from further analysis because they were not robust at the time of the initial assessment in 2012: these are participants with a Kihon Checklist (KCL, Japanese frailty indicator) score of ≥4 or with a phenotype of cognitive impairment (Mini-Mental State Examination score of <22). Therefore, the data of 621 participants were analyzed for changes in robust health statuses between 2012 and 2016 (Figure 1). The study protocol was approved and mandated by the University of Tokyo Life Science Ethics Committee, and informed consent was obtained from all participants prior to participation in the study (Approval number: 12-8). 

### 2.2. Survey Content 

#### 2.2.1. Frailty Score 

Frailty was classified using the 25-point scale questionnaire (KCL) developed by the Ministry of Health, Labor, and Welfare (MHLW) (25 items, α = 0.73). The KCL showed that the questionnaire had satisfactory internal consistency. In the KCL, a score of ≤3 is defined as robust, 4–7 as pre-frailty, and ≥8 as frailty [18]. Meanwhile, in 2016 during the reassessment, participants with KCL scores of ≥4 were classified into the ‘frailty progressing’ group.

#### 2.2.2. Health Literacy

HL was assessed using the method by Ishikawa et al., which was based on five items: (1) the ability to collect accurate health-related information from various sources, (2) ability to extract desired information, (3) ability to understand the obtained information, (4) ability to judge the reliability of the information, and (5) ability to make decisions using the information. Participants were asked for their responses to each question on a 5-point scale: 1 = strongly disagree, 2 = disagree, 3 = neither agree nor disagree, 4 = agree, and 5 = strongly agree [19] (α = 0.86). Low HL was defined as a mean score of ≤3, while a mean score >4 indicated high HL.

#### 2.2.3. Other Variables

In addition to frailty and HL, we assessed the effect of the participants’ mental health [20], social support [21], oral quality of life (oral QOL) [22], and social capital [23] in 2012 on frailty progression.

The Japanese language version of the World Health Organization (WHO)-5 Well-Being Index (score range: 0–25, five items: α = 0.86) was used to assess mental health [24]. Social support was assessed using the Japanese abridged version of the Lubben Social Network Scale (LSNS-6; score range: 0–30, five items: α = 0.82) [25]. Oral QOL was assessed using the Japanese version of the General Oral Health Assessment Index (GOHAI; twelve items: α = 0.89) [26]. Social capital was assessed using three items: (1) cohesion: people in the community are unified, (2) trust: people in the community are trustworthy, and (3) mutual support: people in the community are willing to help their neighbors. These were assessed on a five-point scale: 1 = disagree; 2 = somewhat disagree; 3 = neither agree nor disagree; 4 = somewhat agree; and 5 = agree.

### 2.3. Statistical Analysis

Two-group comparisons were performed as follows: (i) frailty-progressing vs. robust group, based on the frailty status in 2016; and (ii) low vs. high HL in 2012. The Wilcoxon rank-sum test was used for continuous variables, and the χ^2^ test was used for categorical variables to reveal any group differences. Logistic and multiple logistic analyses were used to evaluate factors of frailty progression. Adjusted odds ratios were evaluated using Model 1, with and HL as independent variables. The model was adjusted for age, sex, BMI, education, household status (living with family or not), income, and KCL score in 2012. In addition to all the control variables of Model 1, Model 2 was adjusted for mental health, social support, oral QOL, and social capital. We also compared the variables that influenced frailty progression among HL categories. The significance level was set at *p* < 0.05. Statistical analyses were performed using JMP ver. 13.2.1 (SAS Institute Inc., Cary, NC, USA). 

## 3. Results

### 3.1. Participant’s Baseline Characteristics 

Table 1 summarizes the participants’ baseline characteristics. Of the 621 participants, 144 (23.2%) had low HL. The KCL scores in 2012 and 2016 were 1.5 and 2.6, respectively, with a mean increase in the KCL score of 1.1 over 4 years. In 2016, 463 (74.6%), 138 (22.2%), and 20 (3.2%) participants were classified as robust, pre-frail, and frail, respectively. A total of 158 (25.4%) participants were in the progressing frailty group. Table A1 shows the results of each item of KCL in 2012 and 2016.

### 3.2. Group Comparison 1: Between the Frailty-Progressing Group and Robust Group in 2016

The results of the initial assessment in 2012 indicated that the participants in the frailty-progressing group were older (*p* < 0.001) and had lower HL (*p* < 0.001) than those in the robust group (Table 1). They also had lower scores of mental health (*p* < 0.001), social support (*p* < 0.001), and oral QOL (*p* < 0.001). In addition, the frailty-progressing group had a higher proportion of negative social capital indicators for mutual support (*p* < 0.001). On the other hand, there were no significant differences in gender (*p* = 0.16), BMI (*p* = 0.62), educational background (*p* = 0.56), household status (*p* = 0.53), and income (*p* = 0.68).

### 3.3. Group Comparison 2: Between ‘Low’ and ‘High HL’ Group

Table 2 shows the differences between the low and high HL groups in 2012. There were no differences in age or sex ratio between the two groups. There was no difference in KCL scores in 2012 between the two groups. However, there was a significant increase in the KCL score from 2012 to 2016 (*p* < 0.001) in only the low HL group. In the 2012 assessment, mental health (*p* < 0.001), social support (*p* = 0.02), and mutual aid of social capital (*p* = 0.02) had significantly negative results in the low HL group. The results of each item of HL are shown in Table A2.

### 3.4. Evaluation of Factors Influencing the Progression of Frailty 

The crude and adjusted odds ratios of frailty progression are shown in Table 3. HL showed a significant association in Model 1(adjusted odds ratio [95% confidence interval, CI]: 2.07 (1.32, 3.24) and Model 2 (1.72 [1.07, 2.77]) when confounding factors were introduced. In Model 1, advanced age (1.07 [1.03, 1.12]) and higher KCL score in 2012 (2.19 [1.77, 2.71]) were associated with frailty progression, while male gender (0.51 [0.27, 1.95]) was the factor for the reduced risk of frailty progression. The findings for Model 2 similarly indicated association with frailty progression: advanced age (1.09 [1.04, 1.14]), higher KCL score in 2012 (1.97 [1.57, 2.47]), and negative views toward mutual aid of social capital (2.98 [1.09, 8.14]), while male gender (0.33 [0.17, 0.66]), good mental health (0.90 [0.85, 0.96]), and sufficient social support (0.95 [0.91, 0.98]) reduced the risk of frailty progression. Social capital is no longer significant after adjustment in Model 2.

## 4. Discussion

In this study, we examined the factors that would influence frailty progression over 4 years among healthy community-dwelling older adults who were free from constant nursing care and cognitive impairment. The analytical model was adjusted for age, sex, BMI, education, household status, income, mental health, social support, oral QOL, and social capital indicators. Based on our findings, we observed that HL might be associated with frailty progression in 4 years. 

Among the robust older adults (mean age of 71.6 years in 2012), 22.2% of the population became pre-frail, and 3.2% became frail after 4 years. In a Japanese survey, the overall proportion of frailty for those aged ≥65 years was 11.3%; however, the frailty rate was 7.2% for the 70–74 years age group, [27], which was higher than that in our study population. Furthermore, unlike in other studies, all the participants of this study were robust (KCL score ≤3) as of 2012; this was to focus on the transition to frailty. Therefore, our results suggest that HL in robust older adults may be a determinant of frailty onset and imply the effectiveness of early interventions to improve HL for frailty prevention.

In this study, HL was a determinant of frailty onset and progression, based on the two logistic analysis models and comparison between the robust and frailty-progressing groups in 2016. The results were consistent with those of previous studies that reported an association between HL and frailty [14,15]. For example, Shiraoka et al. assessed the data of 517 community-dwelling older adults with a mean age of 73.2 years in Japan and found that high HL was an independent factor for frailty [14]. According to Nutbeam et al. [28], HL involves three processes including obtaining, understanding and using health information. All these elements are considered to influence people’s behavioral changes. For example, people with low HL are unlikely to participate in cancer screening and other preventive activities [12]. They may also have a higher prevalence of unhealthy habits such as smoking, low physical activity, and insufficient self-care management [7]. Thus, lower HL seems to accompany several negative habits that worsen frailty status in 4 years; however, intervention strategies to improve HL may be useful in preventing, delaying, or slowing the onset of frailty. It is somewhat likely that the participants with higher HL obtained, understood, and used information on frailty and health effectively while the lower HL group may have had some negative habits that exacerbated frailty over the course of 4 years. However, further studies are required to clarify where the differences were in the three processes of HL (i.e., obtaining, understanding, using) between the two groups.

In addition to the logistic analysis that was a direct analysis of the relationship between HL and frailty progression, we performed a comparison to assess the differences between the high and low HL groups in 2012. There were no differences in age, sex, and KCL score in 2012 between the two HL groups, but the low HL group had a significantly higher KCL score in 2016 (Table 2). Various factors cause frailty [3,4]. The KCL is a questionnaire that was originally designed to comprehensively cover multiple aspects of functional decline in activity, exercise, nutrition, oral function, sociability, cognitive capacity, depression in daily life, and mental and physical vulnerabilities [18]. Despite the absence of differences in KCL scores during the initial assessment in 2012, further group analyses revealed that the lower HL group had poorer mental health, less social support, and negative views of social capital of mutual aid; this suggested a significantly faster progression of frailty over 4 years. Previous studies have reported that HL has an intermediary effect on social support and frailty [21,29]. HL has been reported to be an important element to take part in social networks [30]. Network with people promotes health-related information to be obtained, understood, and used through communication, possibly helping to prevent the progression of frailty. The participants in the high HL group may have maintained social connections, which prevented frailty progression. In addition, HL has been considered to favor adaptation to healthy habits such as no smoking, adherence to treatment, and engagement in healthy behaviors [9,15]. Furthermore, a combination of these factors may have affected the findings of the high HL group in our study. However, it is important to note that only KCL data were analyzed during the second assessment in 2016, and the influence of behavioral changes and other factors due to differences in the level of HL requires further investigation. HL can decrease with age due to a decline in reading comprehension, logical thinking, and mathematical ability [31]. Therefore, early interventions to maintain or improve HL will be useful.

In Japan, ‘the community salon project’ has been strongly promoted as part of the policy to provide opportunities for older adults to participate in various activities to live well. Since 2014, it has been advised by the “long-term care prevention promotion support project by community development” that such community salons should be located within 15-min walking distance for older residents [32]. As a result, regional salons have been developed all over Japan, from the very north (i.e., Hokkaido prefecture) to the south end (i.e., Okinawa prefecture), and mutual help and support have been established with great accessibility. It has been reported that HL interventions can be centered on health education to promote healthy behaviors [33]. Our research group has been encouraging peer education programs in “community salons” for those participating citizens to raise awareness of frailty signs. We believe that educational intervention is important to improve the HL of older adults by voluntarily working on self-frailty prevention. These activities are carried out at various community salons sites called ‘local salons’, allowing older residents to freely and conveniently partake in these activities in their communities. These activities are currently being carried out in 80 municipalities in Japan; we believe it will improve HL, promote the prevention of frailty progression in older adults, and, if possible, extend healthy life expectancy at a macro-level.

For future research, it is important to note that existing findings on the association between HL and frailty are controversial [15,18,29,34,35]. This may be explained by the difference in study design, i.e., cross-sectional vs. longitudinal. Another explanation may be the exclusion of non-robust older adults from the initial assessment. It should be noted that in this study, the frailty evaluation is based on a subjective scale (KCL) commonly used in Japan but not globally recognized. Nevertheless, the total score on the KCL is significantly correlated with the global standard of frailty measure, the Cardiovascular Health Study (CHS) (ρ = 0.655, *p* < 0.001) [18]. In addition, the area under the ROC curve is 0.92 as an estimated value of frailty with high sensitivity (89.5%) and specificity (80.7%), therefore proving the sufficient reliability and validity of KCL as a method for frailty evaluation. In terms of evaluation of HL, the current study employed the method based on the previous studies [16,36], but this is different from the three levels of HL defined by Nutbeam [28], distinguished at the more vital level such as the ability to read and understand written information. In developed countries like Japan, the literacy rate is high due largely to the compulsory education system, and mere lack of ability to read written information is unlikely to be a cause of low HL. The selection of appropriate assessment methods for HL should, therefore, take into account the social circumstances of the particular region such as educational systems, socioeconomic statuses, and accessibility to reliable medication. Although HL can affect various behaviors and physical conditions, the study did not thoroughly assess the effects of some individual characteristics. The results indicated that education, income, and household status (i.e., living alone) were also associated with frailty, which is consistent with the results of previous studies [21,37,38]. Further studies are required to determine how these factors influence the progression of frailty, regardless of HL status. 

## 5. Conclusions

In conclusion, for healthy community-dwelling older adults without the need for nursing care and phenotype of cognitive impairment, low HL may be a risk factor for the progression of frailty in four years, regardless of age, sex, basic attributes, socioeconomic factors, and educational background. Older adults with lower HL tended to show negative mental health and lack of social support. It was suggested that effective utilization of social support can be an important approach to improve HL and prevent associated risks of frailty progression. Further, it was suggested that a comprehensive approach to improve HL (education on awareness of frailty signs, exercise, nutrition, and social participation for frailty prevention) might be a useful and effective prevention strategy.

## Figures and Tables

**Table 1:** Comparison between the frailty-progressing group and robust group.

	Total	Frailty-Progressing Group	Robust Group	*p*
N	621	158 (25.4%)	463 (74.6%)	
Age ^a^	71.6 ± 4.6	72.7 ± 4.8	71.2 ± 4.4	<0.001
Sex ^b^				
Male	348 (56.0%)	81 (51.3%)	267 (57.7%)	0.16
Female	273(44.0%)	77 (48.7%)	196 (42.3%)	
BMI ^a^	23.0 ± 2.7	23 ± 2.9	23 ± 2.7	0.62
Educational background ^b^				
Elementary/junior high school graduate	60 (9.7%)	18 (11.4%)	42 (9.1%)	0.56
High school graduate	286 (46.1%)	75 (47.5%)	211 (45.7%)	
University/junior college/college graduate	274 (44.2%)	65 (41.1%)	209 (45.2%)	
Household staus ^b^				
Living with someone	562 (90.5%)	141 (89.2%)	421 (90.9%)	0.53
Living alone	59 (9.5%)	17 (10.8%)	42 (9.1%)	
Income ^b^				
Low	317 (51.5%)	83 (52.9%)	234 (51%)	0.68
High	299 (48.5%)	74 (47.1%)	225 (49%)	
2012 KCL score, mean ± SD ^a^	1.5 ± 1.0	2.0 ± 0.9	1.3 ± 1.0	<0.001
2016 KCL score, mean ± SD ^a^	2.6 ± 2.2	5.7 ± 1.9	1.5 ± 1.0	<0.001
2012–2016ΔKCL score, mean ± SD ^a^	1.1 ± 2.1	3.7 ± 2.0	0.2 ± 1.2	<0.001
Health literacy ^b^				
Low	144 (23.2%)	52 (32.9%)	92 (19.9%)	<0.001
High	477 (76.8%)	106 (67.1%)	371 (80.1%)	
Mental Health: WHO-5 ^a^	19.2 ± 3.6	17.9 ± 3.7	19.7 ± 3.5	<0.001
Social Support: LSNS-6 ^a^	17.3 ± 5.6	15.6 ± 5.7	17.9 ± 5.5	<0.001
Oral quality of life: GOHAI ^a^	56.5 ± 4.6	55.5 ± 4.8	56.8 ± 4.4	<0.001
Social capital indices ^b^				
Trust: People in the community can be trusted.				
1. Disagree/somewhat disagree	15 (2.4%)	6 (3.8%)	9 (1.9%)	0.09
2. Neither agree nor disagree	125 (20.1%)	39 (24.7%)	86 (18.6%)	
3. Agree/somewhat agree	481 (77.5%)	113 (71.5%)	368 (79.5%)	
Cohesion: People in the community are very united				
1. Disagree/somewhat disagree	53 (8.5%)	19 (12%)	34 (7.3%)	0.11
2. Neither agree nor disagree	223 (35.9%)	60 (38%)	163 (35.2%)	
3. Agree/somewhat agree	345 (55.6%)	79 (50%)	266 (57.5%)	
Mutual aid: People in the community are willing to assist their neighbors				
1. Disagree/somewhat disagree	35 (5.6%)	18 (11.5%)	17 (3.7%)	<0.001
2. Neither agree nor disagree	184 (29.7%)	50 (31.8%)	134 (28.9%)	
3. Agree/somewhat agree	401 (64.7%)	89 (56.7%)	312 (67.4%)	

^a^ Assessed using the Wilcoxon rank-sum test; ^b^ Assessed using the χ^2^-test Abbreviations: GOHAI, General Oral Health Assessment Index; KCL, Kihon Checklist; LSNS, Lubben Social Network Scale; WHO-5, World Health Organization-Five Well-Being Index; SD, standard deviation.

**Table 2:** Factors influencing the progression of frailty by health literacy.

	Low HL Group (*n* = 144)	High HL Group (*n* = 477)	*p*
Age ^a^	71.4 ± 4.8	71.6 ± 4.5	0.41
Sex ^b^			
Male	78 (54.2%)	270 (56.6%)	0.61
Female	66 (45.8%)	207 (43.4%)	
2012 KCL score ^a^	1.6 ± 1.0	1.4 ± 1.0	0.20
2016 KCL score ^a^	3.1 ± 2.3	2.4 ± 2.2	<0.001
ΔKCL score ^a^	1.5 ± 2.1	1.0 ± 2.1	<0.001
Mental health: WHO-5 ^a^	18.1 ± 3.8	19.6 ± 3.5	<0.001
Social support: LSNS-6 ^a^	16.4 ± 5.8	17.6 ± 5.6	0.02
Social capital ^b^			
Mutual aid			
Disagree/Somewhat disagree	12 (8.3%)	23 (4.8%)	0.02
Neither agree nor disagree	52 (36.1%)	132 (27.7%)	
Agree/Somewhat agree	80 (55.6%)	321 (67.4%)	

^a^ Assessed using the Wilcoxon rank-sum test. ^b^ Assessed using the χ^2^-test Abbreviations: HL, Health literacy; KCL, Kihon Checklist.

**Table 3:** Multivariate analysis of health literacy for frailty after 4 years.

	Crude Odds Ratio [95% CI]	*p*	Adjusted Odds Ratio [95% CI]: Model 1 ^a^	*p*	Adjusted Odds Ratio [95% CI]: Model 2 ^b^	*p*
Age ^a,b^	1.07 [1.03, 1.12]	<0.001	1.07 [1.03, 1.12]	<0.001	1.09 [1.04, 1.14]	<0.001
Sex (Male) ^a,b^	0.77 [0.54, 1.11]	0.16	0.51 [0.27, 0.95]	0.04	0.33 [0.17, 0.66]	<0.001
BMI ^a,b^	1.01 [0.94, 1.08]	0.81	1.01 [0.94, 1.09]	0.74	1.02 [0.94, 1.1]	0.68
Educational background ^a,b^						
Elementary/junior high school graduate	1.38 [0.74, 2.56]	0.31	1.06 [0.52, 2.17]	0.88	1.04 [0.48, 2.23]	0.92
High school graduate	1.14 [0.78, 1.68]	0.49	1.07 [0.69, 1.64]	0.78	1.04 [0.66, 1.64]	0.87
Household status (not living with family) ^a,b^	1.21 [0.67, 2.19]	0.53	0.98 [0.5, 1.92]	0.95	0.92 [0.45, 1.88]	0.82
Income (low) ^a,b^	1.08 [0.75, 1.55]	0.68	0.68 [0.36, 1.27]	0.22	0.58 [0.30, 1.11]	0.10
2012 KCL Score ^a,b^	2.16 [1.76, 2.64]	<0.001	2.19 [1.77, 2.71]	<0.001	1.97 [1.57, 2.47]	<0.001
Health literacy (low)	1.98 [1.32, 2.96]	<0.001	2.07 [1.32, 3.24]	<0.001	1.72 [1.07, 2.77]	0.02
Mental health: WHO-5 ^b^	0.88 [0.83, 0.92]	<0.001			0.90 [0.85, 0.96]	<0.001
Social support: LSNS-6 ^b^	0.93 [0.9, 0.96]	<0.001			0.95 [0.91, 0.98]	0.01
Oral Quality of Life: GOHAI ^b^	0.94 [0.91, 0.98]	<0.001			0.99 [0.94, 1.03]	0.53
Social capital indices ^b^						
Trust: People in the community can be trusted.						
Disagree/Somewhat disagree	2.17 [0.76, 6.23]	0.15			0.98 [0.26, 3.75]	0.98
Cohesion: People in the community are very united						
Disagree/Somewhat disagree	1.88 [1.02, 3.48]	0.04			0.54 [0.21, 1.38]	0.20
Mutual aid: People in the community are willing to help their neighbors						
Disagree/Somewhat disagree	3.71 [1.84, 7.5]	<0.001			2.98 [1.09, 8.14]	0.03

Assessed using logistic analysis and multiple logistic analyses: dependent variable (frailty progression) and independent variable (HL) ^a^ Model 1: Adjusted for age, sex, BMI, education, household status (living with family or not), income, 2012 baseline KCL score. ^b^ Model 2: Age, sex, BMI, education, household status (living with family or not), income, 2012 baseline KCL score, mental health, social support, oral quality of life, and social capital (trustworthiness of community members, cohesion of community members, and mutual support of community members) Abbreviations: CI, confidence interval; GOHAI, General Oral Health Assessment Index; KCL, Kihon Checklist; LSNS, Lubben Social Network Scale; WHO-5, World Health Organization-Five Well-Being Index.

## Data Availability

The data in this study is restricted because the ethical approval was not sought for public data sharing from the Ethics Committee at the University of Tokyo and the participants were not informed of possible public data sharing when they provided informed consent. However, data can be made available from a non-author of contact at Institute of Gerontology, the University of Tokyo (contact via info.frail@iog.u-tokyo.ac.jp) for researchers who meet the criteria for access to confidential data.

## Appendix A

**Table A1:** 2012 and 2016 KCL applicable ratio.

No	Questions		Answer	
			2012	2016
1	Do you go out by bus or train by yourself?	NO	15 (2.4%)	18 (2.9%)
2	Do you go shopping to buy daily necessities by yourself?	NO	9 (1.4%)	15 (2.7%)
3	Do you manage your own deposits and savings at the bank?	NO	20 (3.2%)	67 (10.8%)
4	Do you sometimes visit your friends?	NO	133 (21.4%)	239 (38.5%)
5	Do you turn to your family or friends for advice?	NO	20 (3.2%)	81 (13.1%)
6	Do you normally climb stairs without using handrail or wall for support?	NO	74 (11.9%)	125 (20.2%)
7	Do you normally stand up from a chair without any aids	NO	21 (3.4%)	37 (6.0%)
8	Do you normally walk continuously for 15 min	NO	12 (1.9%)	56 (9.0%)
9	Have you experienced a fall in the past year	YES	70 (11.3%)	153 (24.6%)
10	Do you have a fear of falling while walking	YES	101 (16.3%)	128 (20.7%)
11	Have you lost 2 kg or more in the past 6 months	YES	53 (8.5%)	109 (17.6%)
12	Height: cm, weight: kg, BMI: kg/m^2^ (If BMI is less than 18.5, this item is scored.)	YES	31 (5.0%)	142 (22.8%)
13	Do you have any difficulties eating tough foods compared to 6 months ago?	YES	48 (7.7%)	87 (14.0%)
14	Have you choked on your tea or soup recently	YES	76 (12.2%)	99 (15.9%)
15	Do you often experience having a dry mouth	YES	111 (17.9%)	165 (26.6%)
16	Do you go out at least once a week	NO	23 (3.7%)	87 (14.0%)
17	Do you go out less frequently compared to last year	YES	67 (10.8%)	140 (22.5%)
18	Do your family or your friends point out your memory loss?	YES	42 (6.8%)	94 (15.2%)
	e.g., “You ask the same question over and over again.”			
19	Do you make a call by looking up phone numbers	NO	39 (6.3%)	85 (13.7%)
20	Do you find yourself not knowing today’s date	YES	99 (15.9%)	100 (16.1%)
21	In the last 2 weeks have you felt a lack of fulfilment in your daily life?	YES	36 (5.8%)	42 (6.8%)
22	In the last 2 weeks have you felt a lack of joy when doing the things you used to enjoy?	YES	8 (1.3%)	23 (3.7%)
23	In the last 2 weeks have you felt difficulty in doing what you could do easily before?	YES	32 (5.2%)	74 (12.0%)
24	In the last 2 weeks have you felt helpless	YES	29 (4.7%)	39 (6.3%)
25	In the last 2 weeks have you felt tired without a reason	YES	34 (5.5%)	66 (10.7%)
	BMI, body mass index; KCL, Kihon Checklist.			

## Appendix B

**Table A2:** Average value of HL item.

No	Questions	Mean ± SD
1	Seeking information from various sources	4.3 ± 0.7
2	Extracting relevant information	4.2 ± 0.7
3	Considering the credibility of the information	4.0 ± 0.6
4	Understanding and communicating the information	4.1 ± 0.6
5	Making decisions based on the information	4.1 ± 0.7
	HL, Health literacy; score range (1–5)	
